# Boys and Girls in Secondary Schools

**Published:** 1923-02

**Authors:** 


### DIFFERENTIATION IN SECONDARY SCHOOLS.

THE Consultative Committee of the Board of Education on Differentiation of Curricula for Boys and Girls in Secondary Schools has just issued a report (Stationery Office 2/9 net), which should be as valuable to the medical man as to the educationist. To the non-medical man, indeed, it will present much that is new and of great interest, while the doctor will turn at once to the valuable memorandum drawn up by Dr. Adami, Vice-Chancellor of Liverpool University, the only medical man on the Committee, on anatomical and physiological differences between the sexes, a subject of obvious moment alike to educationists and to masters of medicine. While finding it easy enough to elicit opinions from the witnesses, the Committee experienced singular difficulty in eliciting a body of well-established facts, and in a sense the inquirers found themselves at the parting of the ways. Some of the champions of equal rights and privileges for the two sexes still maintained their old attitude of minimising the differences between them. Others, realising that victory has been gained, and that girls now are given educationally the same opportunities as boys, are ready to recognise, in the words of the introduction, that "equality does not demand identity, but is compatible with, and even depends upon, a system of differentiation under which either sex seeks to multiply at a rich interest its own particular talents."

### The Over-pressure of Girls.

The Committee make the following "recommendations for differentiation between boys' and girls' schools," in which the health aspect of school life is predominant:—

That girls should, as a rule, be encouraged to take the First School Examination about a year later than boys; and that if and when State Scholarships are again awarded, the regulations for girl candidates should be modified accordingly.

That in girls' schools the pressure of external examinations, which is in our opinion partly responsible for much over-teaching and for the unduly passive attitude of many pupils, should be reduced wherever possible.

That more attention should be devoted by parents, head mistresses and school doctors to the possibility of taking suitable precautions for the protection of girls against physical fatigue and nervous overstrain.

That in girls' day schools and in other day schools attended by girls steps should be taken to reduce the amount of preparation required from girls, which, in some instances, is at present excessive in view of the relatively heavy domestic duties often performed by them in their homes.

That the morning session in girls' schools should not exceed three-and-a-half hours.

### Need for Further Inquiry.

There is still so much that we do not know on these subjects that their investigation would seem to be very far from nearly a conclusion. Thus the Committee recommend further:—

That systematic inquiries should be undertaken in order to collect trustworthy data on the question of the relative susceptibility of boys and girls between the ages of 11 and 18 (or 19) to mental and physical fatigue, both in ordinary school work and in games.

That further inquiries should be undertaken with a view to ascertaining what games and physical exercises are most suitable for girls of varying ages, more especially day girls, in the different types of schools.

That research should be undertaken by psychologists and teachers on groups of boys and girls respectively, drawn from Secondary Schools of different types, with a view to collecting data in regard to the intellectual and emotional differences between the sexes in their bearing on education.

